# Herpesviruses: latency and reactivation – viral strategies and host response

**DOI:** 10.3402/jom.v5i0.22766

**Published:** 2013-10-25

**Authors:** Bjørn Grinde

**Affiliations:** Department of Mental Health, Norwegian Institute of Public Health, Oslo, Norway

**Keywords:** herpes simplex, Epstein–Barr, cytomegalovirus, varicella zoster, reemergence, immune defense, viral pathology, oral cavity

## Abstract

Eight members of the *Herpesviridae* family commonly infect humans, and close to 100% of the adult population is infected with at least one of these. The five that cause the most health concerns are: herpes simplex virus (HSV) type 1 and 2, Epstein–Barr virus (EBV), cytomegalovirus (CMV), and varicella zoster virus (VZV). In addition, there are human herpes virus (HHV) types 6–8. The review starts by introducing possible viral strategies in general. The particular biology and host relationship of the various human herpesviruses, including their pathology, are examined subsequently. Factors that contribute to the maintenance of latency and reactivation of viral replication are discussed. There will be special reference to how these viruses exploit and contribute to pathology in the oral cavity. Reactivation does not necessarily imply clinical symptoms, as reflected in the asymptomatic shedding of EBV and CMV from oral mucosa. The immune response and the level of viral output are both important to the consequences experienced.

Viral infections are generally recognized only when they cause, or are suspected to cause, disease. The more common scenario is most likely that viral activity within a host does not result in any clinical symptoms. This point has been demonstrated for a range of potentially pathogenic viruses by finding a high prevalence in healthy individuals (1, 2). It is important to keep this in mind when exploring these agents and their relationship with the host.

When symptoms do occur, they can be classified into two groups. In the first group, symptoms are induced by the virus for the particular purpose of advancing survival and propagation. Typical examples are related to the transfer of viral particles between individuals, as the production of viral particles does not necessarily require any noticeable damage to the host. Coughing, diarrhea, and cold sore represent viral strategies that presumably evolved for the purpose of contagion. In the second group, symptoms are caused by the host response or inadvertently by excess viral activity. When the immune system reacts to foreign antigens, there will be collateral damage. In some cases, however, the immune system appears to overreact causing potentially more harm than the virus itself. The response to certain strains of influenza virus is a possible example (3).

Viruses that have a long-term evolutionary relationship with their host species are normally relatively benign. These viruses rely on their hosts for replication, thus evolution has designed the interaction to run smoothly. It is generally not in the evolutionary interest of viruses to kill or seriously maim their residence, as that would impede further progress. Consequently, the more dangerous viruses are those with a recent zoonotic history; such as HIV, SARS, Ebola, and avian influenza. The human herpesviruses most likely have a long evolutionary history of coevolution with our species. Thus, in a normal environment, a healthy individual will rarely experience their presence. The main exception being symptoms related to viral transmission, such as chickenpox blisters and herpes cold sores. The point of viral restraint is particular relevant for herpesviruses due to their strategy of latency. Latent viruses require a host who stays alive and is healthy enough to interact with others.

There are two known examples of zoonotic herpesvirus infections: the cercopithecine herpesvirus-1 and the murine gamma herpesvirus. The former has a very high fatality rate due to encephalomyelitis but is fortunately rare (4); the latter is more common, one study suggests a prevalence of 4.5% in a general population, but it is relatively benign (5). This review focuses on the human herpesviruses. Although all eight subtypes are discussed, occasionally the focus will be on one or two representative examples. This constraint is required for brevity but also reflects limits of the present knowledge.

Viruses can be divided into three types relating to their replicative strategies. Latency with occasional reemergence is one. Viral latency should not be confused with clinical latency, which is the incubation period in which the virus is active, but symptoms have not yet appeared. A second option is the ‘hit-and-run’ approach. Influenza and the diarrhea causing norovirus are typical examples. They display a rapid burst of replication but are subsequently cleared from the system. They depend on their hosts to shed large quantities of viral particles in the hope that some will find the way to novel individuals. The third option is the ‘slow-and-low’ tactic. These viruses replicate continuously, but at a level sufficiently low not to seriously damage their host or to provoke an immune response of sufficient magnitude to risk expulsion. Human spumavirus (6) and torque teno virus (a member of *Circoviridae* that infects humans) (7) appear to follow this strategy. There is no clear-cut distinction between latency and the slow-and-low approach. Even typically latent viruses are not necessarily 100% dormant in-between outbreaks, and in both cases viral activity will fluctuate. Whether the virus follows a slow-and-low strategy, or has cycles of reactivation, the number of viral particles present is defined by the balance between viral proliferation and the capacity of the immune system for clearance. If this balance moves too far in the direction of viral particles, adverse consequences are likely to occur.

Torque teno viruses are not known to cause clinical symptoms, yet they are present in the vast majority of adults (7). The important question is why herpesviruses occasionally are responsible for considerable pathogenicity. The problem is likely related to the way they are designed for reactivation from latency; that is, rather than a slow-and-low strategy, they have bursts of more vigorous replicative activity. Understanding what regulates the level of viral productivity, and the immune response, may help us find strategies to deal with the clinical problems.

## Herpesviruses – biology and pathology

**Fig. 1:**
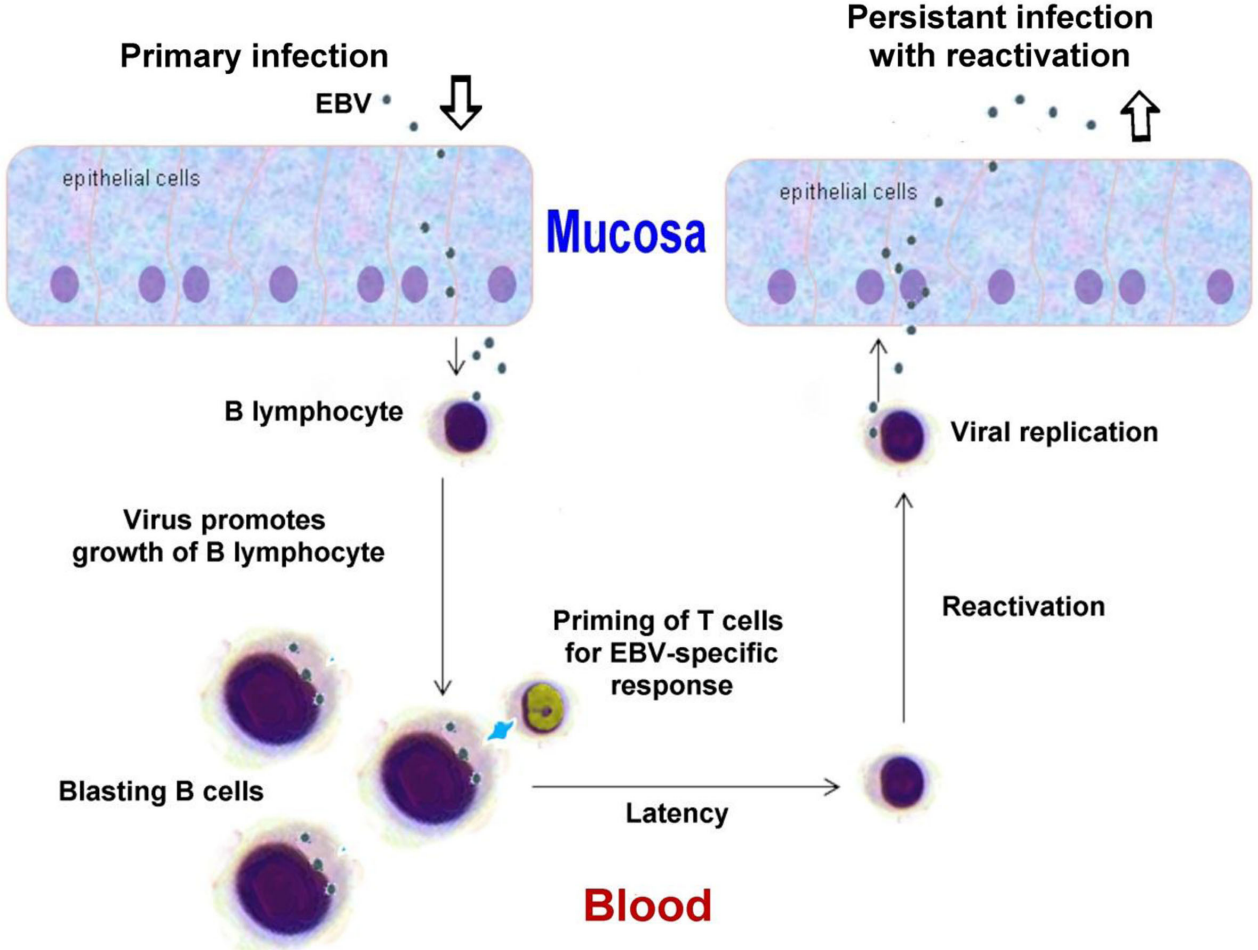
Schematic diagram of EBV replication cycle as an example of a typical herpesviral strategy. Modified from a figure in Wikimedia Creative Commons, author Graham Colm.

The human herpesviruses are large (typically 100–200 nm), enveloped (i.e. membrane-bound) viruses. They contain double-stranded DNA genomes packed in an icosahedral protein cage. The various genomes include 70–200 predicted open reading frames, thus as to gene diversity they are among the most complex human viruses. Moreover, the number of open reading frames grossly underestimates the true genomic output. For example, in the case of cytomegalovirus (CMV), alternative splicing and optional start codons give rise to more than 700 different viral proteins (8). In addition, there are a large number of non-coding RNAs based on the viral DNA that serve various regulatory functions. In Fig. 1 is shown the lifecycle of Epstein–Barr virus (EBV) as a typical example of a herpesviral strategy.

The large repertoire of herpes genes allows for an intricate relationship with the host. Not only do these viruses manipulate the cells they infect but also the immune response.

The *alpha* subfamily (varicella zoster virus [VZV] and herpes simplex virus [HSV]-1 and -2) primarily target neurons for long-term residency, but they also replicate in epithelia, which is essential for efficient transmission through skin or mucosa. The others (*beta* and *gamma* subfamilies) prefer various subsets of leukocytes, but most of them can also infect epithelial cells. Cell tropism is determined by the presence of cell surface receptors, as well as by whether the intracellular conditions are supportive for viral activity. In general, the replication takes place within the nucleus. In some host cells, gene products based on latency-associated transcripts accumulate, which lead to a period of latency. If transcription is shifted to lytic products, novel viral particles are produced, which typically leads to the death of the cell. The reactivation is traditionally associated with malaise but does not necessarily lead to any notable symptoms.

Below are outlined the main clinical outcomes for the various viral types. An overview of the viruses is shown in Table 1. A review of the management of pathology has been recently published (9).

**Table 1:** Key properties of human herpesviruses

Trivial name and acronym	Formal name	Type	Oral affection	Other pathology	Primary target cells	Main sites of latency
Herpes simplex virus-1 (HSV-1)	Human herpesvirus 1	Alpha	Cold sores (herpes ulcers)	Genital ulcers, related skin lesions, keratitis, encephalitis, meningitis	Mucoepithelia	Sensory and cranial nerve ganglia
Herpes simplex virus-2 (HSV-2)	Human herpesvirus 2	Alpha	Cold sores (herpes ulcers)	Genital ulcers, as HSV-1 but more rare	Mucoepithelia	Sensory and cranial nerve ganglia
Varicella zoster virus (VZV)	Human herpesvirus 3	Alpha	Possible oral manifestation of chicken pox and herpes zoster	Chicken pox, herpes zoster	Mucoepithelia	Sensory and cranial nerve ganglia
Epstein–Barr virus (EBV)	Human herpesvirus 4	Gamma	Hairy leukoplakia, periodontitis, nasopharyngeal carcinoma	Mononucleosis, lymphoma	Epithelial and B-cells	Memory B-cells
Cytomegalovirus (CMV)	Human herpesvirus 5	Beta	Periodontitis?	Mononucleosis	Monocytes, lymphocytes and epithelia	Monocytes, lymphocytes
Roseola virus (HHV-6)	Human herpesvirus 6A and 6B	Beta		Roseola in infants	T-cells	Various leukocytes
Roseola virus (HHV-7)	Human herpesvirus 7	Beta		Roseola in infants	T-cells	T-cells, epithelia
Kaposi's sarcoma-associated virus (HHV-8)	Human herpesvirus 8	Gamma		Kaposi's sarcoma	Probably lymphocytes and epithelia	B-cells

### Herpes simplex virus

HSV-1 is primarily associated with blisters, referred to as cold sores, or *herpes labialis*, on the lips. The HSV-2 is associated with related genital sores or blisters; however, both viruses can cause lesions at either site. In fact, the HSV-1 is today the most common form found in genital herpes (10). Sores typically occur a few days after the primary infection, and reappear more or less regularly later in life for a substantial percentage of those infected. The blisters contain abundant viral particles and presumably represent a main strategy for viral transmission. Host factors, such as the intensity of the immune response, may worsen the symptoms. The viruses take life-time residency in nerve cells and are transported to the mucosa along axons. Over time, the episodes tend to diminish in frequency and severity. The genital form is less likely to cause recurrent blisters, but the virus may still be shed through the mucosa.

The mucosal sores are the common sign of an active infection, but HSV can also produce cutaneous lesions, particularly around the nails of fingers and toes, a condition referred to as *herpetic whitlow* (11). In the absence of gloves, it used to be a common problem for dental workers (12). HSV can also reach the eyes causing keratitis, which may lead to blindness (13). Based on their affinity for neurons and epithelial cells they may attack the brain resulting in encephalitis or meningitis (14). Whereas the mucosal and cutaneous symptoms have obvious significance for viral transmission, the activity in the brain appears to be an incongruity. It may reflect stress factors in the host causing demoted immune surveillance.

### Varicella zoster virus

The VZV is related to the HSV both in evolutionary terms and in cell tropism and, as might be expected, the clinical picture has shared characteristics. The primary infection with VZV normally results in chickenpox. The disease is typically accompanied by malaise, such as low-grade fever, nausea, aching muscles, and headache. It starts with a vesicular, itchy rash that primarily affects the trunk and the head. VZV may also form ulcers in the oral cavity.

The clinical picture is somewhat different if the virus is reactivated later in life. The disease is then referred to as *herpes zoster* or shingles. In both cases, the virus causes skin rash with blistering, but in the recurrent form typically on a more limited area of the body. Apparently, the immune system prevents a more global viral activity but is unable to avert a limited number of nerve cells from producing virus that are brought to the terminating area of their axons; that is, to a particular dermatome (15). As in the case with HSV, VZV is also known to cause encephalitis.

### Epstein–Barr virus and cytomegalovirus

EBV and CMV belong to respectively the *gamma* and *beta* subfamily of *Herpesviridae*, yet the clinical picture is related. EBV primarily infects B-cells, whereas CMV is designed to enter monocytes besides lymphocytes. Both of them also infect epithelial cells. They cause mononucleosis, or mononucleosis-like symptoms, although the condition is more commonly associated with EBV. The illness is most prevalent in adolescents, where it is referred to as kissing disease based on a typical route of transmission. The condition manifests itself with fever, sore throat, fatigue, and swollen lymph nodes. Presumably, it is a consequence of the inability of the immune system to handle the virus in an appropriate fashion at this stage of life. Those who first encounter these viruses as infants rarely develop symptoms.

Congenital infection with CMV is one of the leading viral causes of birth defects (16). EBV, however, is known to be tumorigenic. It is associated with various forms of lymphomas as well as with nasopharyngeal carcinomas. In immunocompromised individuals, particularly in association with AIDS, the virus may cause oral hairy leukoplakia, a condition characterized by white patches typically on the lateral surface of the tongue (17).

If the host is unable to keep these viruses at bay, they may also contribute to periodontitis (18, 19). This point has been indicated by the successful treatment of a patient, suffering from severe periodontitis, with valacyclovir (20). The patient did not respond to traditional treatment, and prior to the use of valacyclovir there were exceptionally high levels of EBV in affected periodontal pockets, which disappeared following 10 days of medication.

### Human herpesvirus -6, -7 and -8

Human herpesviruses (HHVs)-6–8 were discovered relatively late. As with the other herpesviruses, at least types 6 and 7, often referred to as roseola viruses, can form skin lesions; in this case referred to as *exanthema subitum* or *roseola* *infantum* (21). The condition is usually seen only in infants less than 2 years of age. The onset is characterized by sudden high fever. As the fever subsides, a red rash appears. It usually begins on the trunk, but spreads to the legs and neck. Although only some 30% develop symptoms, the vast majority of infants 2 to 4 years old harbor the virus (21).

HHV-8 is associated with Kaposi's sarcoma, a form of cancer that became known through its diagnosis in patients with AIDS (22). The virus is common in sub-Saharan Africa but rarer in other parts of the world. Presumably, the majority of those infected show no symptoms.

## Viral strategy

### An act of balance

The emerging picture, of the natural biology of herpesviruses, is a primary infection with mild or no symptoms, and a highly successful establishment of a long-term relationship with the host. Viral activity may be associated with lesions either in the skin, the oral cavity, or genital region as a means of transmitting viral particles to novel hosts. The normal course of host–viral relationship implies a well-regulated viremia and thus limited malaise. However, certain factors cause this evolutionary-designed, benign relationship to fault.

For one, the design is tuned to a ubiquitous presence of viruses in the population, and the acquisition of virus at an early age. As such, they may be referred to as part of a normal, microbiotic flora; although in ancient times all subtypes were probably not present in all human subpopulations. An elevated level of hygiene in industrialized societies restricts this early transmission. When people are affected at a later stage in life, the immune system has taken on a somewhat different quality. The resulting misbalance of viral activity may cause diseases, of which mononucleosis presumably is a typical example. Not only do these patients experience more malaise than infected infants but they are also at higher risk for recurrent problems later in life (23).

The balance between viral activity and immune suppression may also be compromised if the quality of immune surveillance is reduced, particularly in cases of a depressed T-cell immune function. This is a problem for people who suffer from innate or acquired immune deficiency, the latter exemplified by AIDS. Patients who receive organ transplants are generally given immunosuppressant medicines that produce a related effect (24). Physical or psychological stress (25), and the reduction in bodily functions associated with aging (26), may be sufficient to disturb the delicate balance between viral activity and immune response.

In addition to the problems related to increased viral load, some herpesvirus, notably EBV and HHV-8, are associated with cancer. Most viruses that reproduce in the nucleus require the presence of host factors associated with replication and transcription; thus viral replication is, to a lesser or greater extent, dependent on mitotic activation of the cell they inhabit. Moreover, the herpesviruses manufacture gene products aimed at helping the cell survive; an effect that is at least partly due to microRNAs produced from the viral genome and acting as regulators of cellular genes (27). EBV in particular produces factors in the early phase of infection that lead to the immortalization of B-lymphocytes (24). This strategy of stimulating the cell requires a fine balance. If the physiology of the cell tips too far in the direction of growth, the result may be uncontrolled cell division, that is, cancer. The problem is rarely an issue for viruses that target neurons, as these cells are more permanently left in a non-replicative state. However, the lymphocytes and epithelial cells are more tuned toward replication; and consequently more in danger of having the balance meant to control cell growth overthrown. It should be noted that compared to the prevalence of the herpesviruses, this unfortunate event (for both host and virus) is rare.

### The role of the oral cavity

The oral cavity plays a vital role in the transmission of a large range of viruses including most human herpesviruses. The crucial issue seems to be that the oral mucosa has certain preferred features compared to skin or genital mucosa: The mouth offers more efficient transmission because the virus can be dispersed in aerosols, either released by normal breathing, but more efficiently produced upon coughing or spitting. Moreover, as enveloped viruses, the herpes family requires moisture for survival. The blisters on the skin serve the purpose as long as they are watery, but the virus relies on skin-to-skin contact with another person, or immediate contact between objects touched by the infected person and the next host. The occurrence of kissing and food sharing by mouth contribute further to the role of the human mouth in the transmission of pathogens. Biting is still considered to be a relevant risk factor in the transfer of viruses from other primates (28).

Viruses such as EBV and CMV are common in the oral mucosa (18, 19), but also HSV, HHV-6 and HHV-7 can be shed from a healthy oral mucosa (24). Apparently, all herpesviruses have evolved the capacity to access the oral cavity. EBV, CMV, and HHV-6–8 replicate in epithelium cells or various leukocytes where the latter can infiltrate the mucosa. VZV, and HSV-1 and -2 are latent in neurons, but the viral particles are transported via axons to mucosa where the virus may continue replicating in epithelial cells.

It should be noted that using the mouth as part of the strategy for viral contagion does not require the formation of sores, blisters, or other visible changes in the mucosa. However, clinical symptoms associated with the mucosa generally imply a higher abundance of viral particles, and thus a boosted chance of transmission.

## Latency and reactivation

### Features associated with latency and reactivation

Reactivation is a dangerous option for the virus. An active replication will tend to induce various host mechanisms, involving either the immune system or internal signaling in the cell, leading to the death of the infected cell. For every viral genome that successfully produces infective progeny, several viruses probably initiate this process, but fail at some point along the way.

In order to establish latency, the viral genome is circularized to form an episomal DNA element packed in histones (29). For lytic activation to occur, the genome must be linearized (30). During latency, the viral DNA is copied by cellular DNA polymerases, along with the chromosomes, preferably when the cell engages in mitosis. This contrasts with lytic replication in which the viral DNA polymerase is engaged, reflecting a viral takeover of the cell. For latency, the virus relies on the host's epigenetic mechanisms for the silencing of viral genes, such as packaging of the DNA in particular types of histones and specific methylation programs (30). Presumably it is safer to keep the production of viral proteins to a minimum during latency in order to avoid immune surveillance; but at the same time it is important for the virus to be copied in order to maintain long-term presence, as some of the daughter cells will eventually die.

The viral genome is not completely silenced. Latent EBV, for example, expresses a small portion of its genes (31). The virus exhibits three different latency programs, each comprising a limited and distinct set of viral proteins and RNAs (32, 33). Upon infecting a resting B-cell, the virus starts with the more comprehensive latency III program. The resulting proteins induce the B-cell to proliferate. The virus subsequently gradually shuts off genes, entering latency II and eventually latency I. In latency I, only one protein and some non-coding RNAs are expressed. The protein, EBNA-1, binds to a replication origin in the viral genome and is instrumental in securing synthesis of DNA when the host cell divides.

Lytic gene products are also produced in three consecutive stages: immediate-early, early, and late (23). The primary role of the immediate-early lytic products is to function as transactivators, enhancing the expression of later lytic genes. The early lytic products take on more diverse functions, such as replication, metabolism, and blockade of antigen processing. The late lytic products are typically proteins with structural roles, including the units of the capsid and glycoproteins that are incorporated in the viral envelope. Other late gene products, such as BCRF1, help EBV evade the immune system. The actual changes in both viral and host cell transcription and translation over the various stages of viral latency and reactivation are highly complex, as demonstrated with various functional genomics studies on CMV (34).

Herpesviruses, being enveloped, normally bud from the cell membrane, which implies that lysis of the cell is not required. However, the takeover necessary for running active production of viral particles in epithelial cells or leukocytes typically results in cell death.

In the case of HSVs, much of our knowledge of the molecular control of latency comes from studies with virus in cultured neuronal cells (30). Here axonal transport plays an important role. If the virus first enters at a distant spot on an axon, as compared to closer to the cell body, the situation favors latency. The explanation may be inefficient axonal transport of virion-associated regulatory factors, such as the HSV lytic initiator protein VP16 (35). The protein is released from the viral particle upon entry, and subsequently requires independent transport to the nucleus in order to initiate the replicative program. Thus, if the distance to the cell body is large, less VP16 will reach the nucleus, and the onset of viral productivity is compromised. Later on, other factors may initiate de novo synthesis of VP16 in the nucleus thus causing reactivation.

### Viral impact on immune surveillance

Herpesviruses are known for their ability to establish lifelong infections. In order to do so they require a strategy for immune evasion, consequently the viruses have evolved a variety of ways to manipulate the immune system of the host. One typical example is based on molecular mimicry. Most of the viruses encode homologs of cellular interleukins (IL), chemokines, or chemokine receptors (36). The EBV gene BCRF1, for example, encodes a viral homolog of human IL-10 (37). The viral version of IL-10 impairs NK cell mediated killing of infected B-cells, interferes with CD4+ T-cell activity, and modulates cellular cytokine response. Another strategy for immune evasion is to reduce the presentation of viral antigens via the major histocompatibility complex (MHC) of infected cells (38). Some viruses simply downregulate or inhibit the display of both MHC class I and II molecules. The EBV-encoded BNLF2a gene reduces antigen presentation, and consequently recognition by CD8+ T-cells, in newly infected cells (37).

The manipulation of the immune system offers the reactivated virus at least partial relief from immune surveillance. It is, however, a feature that increases the risk of the balance tipping toward excessive viral productivity, which is not in the interest of the virus as it can lead to death or serious disability of the host. Evolution apparently has balanced this possibility, leading to a situation where the viral activity in a normal host is limited to a considerable extent by immune surveillance. The point is substantiated by the observation that compromised immunity, as in the case of patients receiving immune suppressants, often lead to a drastic increase in viral activity (24).

Other data suggest that herpesviruses can in fact form a symbiotic relationship with their hosts. In a mouse model, it has been shown that the systemic activation of macrophages and the prolonged production of interferon-gamma initiated by herpesviral infections protect against subsequent disease caused by the highly pathogenic bacteria *Listeria monocytogenes* and *Yersinia pestis* (39). The suggestion of symbiosis is plausible as it is obviously not in the interest of the virus to have their host succumb to other infections.

### External factors involved in reactivation

In animal models, and most likely in humans as well, reactivation of various herpesviruses can be induced by local trauma (e.g. in the form of surgery) or systemic stress. An example of the latter is to elevate the body temperature of mice to 43^o^C for 10 min (40). The appearance of HSV cold sores correlates with a wide range of stressors, including mental tension, fatigue, and exposure to bright light (41). More than 100 years ago it was shown that applying trauma to a nerve, for example in connection with the treatment of chronic pain, can lead to an outbreak of herpes in the dermatome associated with the nerve (30).

Other cell stressors, such as transient interruption of protein synthesis or hypoxia, are sufficient to induce viral activity – an effect that may be mediated by the disruption of mTOR kinase activity (42). This enzyme has a central role in responding to nutritional or to stress-related cellular events by impacting on mRNA translation. Apparently, in the case of HSV, it is sufficient to inhibit mTOR (by chemical means) in the distal part of an axon; a signal causing viral reactivation is then sent to the cell body harboring the viral episome. Presumably, a skin trauma affecting the nerve endings of infected neurons may cause a similar reactivation. In order to maintain latency, the neuron must be functional, active, and healthy. Similar to rats, the virus prepares to ‘leave the ship’ if it is likely to ‘sink’.

In cell culture systems, it is also possible to induce reactivation by interfering chemically in ways that impact on gene activity, using compounds that for example block histone methylation or appropriately designed interfering RNAs (30). The question is what factors cause a similar impact on gene expression *in vivo*. As to HSV, it is known that environmental triggers such as emotional stress, fever, UV exposure, hormonal changes, dental surgery, and cranial trauma can cause activation (30); but it is not known whether these stimuli act directly on the infected neuron, or indirectly by means of bodily functions.

One possible mechanism for the effect of mental stressors is via virus-specific CD8+ T-cells. These cells are often found in association with infected neurons, sometimes connected to the neuron via immunological synapses. They produce interferons and related factors that presumably contribute to the maintenance of latency and at the same time help the neuron survive (43). Both mental and physical stressors are known to influence the activity of CD8+ T-cells through the release of neuroendocrine factors, a mechanism that may link the control of HSV latency to activity in the sympathetic nervous system (44). In the case of EBV, the latent virus harbored in B-cells can be reactivated *in vitro* by stimulating B-cell receptors, suggesting that reactivation *in vivo* may occur when the infected B-cell responds to unrelated infections (23). The point may help explain why reactivation of EBV occasionally appears as a secondary infection.

The aging immune system is no longer able to control the virus efficiently leading to a more chronic, slow-and-low rather than latent type of infection (45). A range of deteriorative immunological changes are expected to correlate with aging, but it is not known exactly what causes the concomitant increase in herpesvirus activity. In fact, the stress associated with space flight is sufficient to cause reactivation of latent herpesviruses, presumably by downregulating cellular immunity (46); which suggests that even a minor decline, or change, in immunological function may be enough. This point is also reflected in the observation that it makes considerable difference whether the individual is first infected as an infant or in puberty, as exemplified by the case of EBV and mononucleosis. One probable cause of an increase in EBV activity late in life is actually a prior increase in CMV activity. The latter event results in an expansion of senescent CD8+ T-cells which lack CD28, but are directed at CMV epitopes; an expansion that has been suggested to restrict immune response to other pathogens including EBV (47). In other words, when CMV (and possibly other herpesviruses) gradually produces more viral proteins and particles, the concomitant attempt of counteracting the situation by an aging immune system can demote the capacity to fight these viruses.

## Concluding remarks

We have considerable knowledge of the various pathways of molecular signaling that can lead to reactivation, and we have empirical information of environmental triggers doing the same. What we do not know is which molecular or cellular pathways the environmental factors use. It could be through some of the options outlined by, mostly *in vitro*, experiments; or it could be novel mechanisms. Apparently, viral activity depends on a delicate balance of constraining and activating factors. Minor disturbances that upset this balance seem sufficient to lead the virus toward production of progeny, and presumably this disturbance can result from a variety of effectors.

In most cases, reactivation does not lead to serious disease. It is sufficient for the virus to be shed in the oral cavity, and even HSV appears in the saliva in the absence of sores (48). Then again the level of viral activity most likely correlates with clinical symptoms. Innate and acquired host factors will affect the balance between viral activity and immune surveillance, making some people more susceptible to problematic infections than others.

In this context, it should be mentioned that we tend to attribute guilt by association. If symptoms correlate with the detection of virus, we tend to assume that the virus is responsible. This may lead to a faulty diagnosis and suboptimal treatment. The herpesviruses are likely to be reactivated as a consequence of a variety of conditions, but they are not necessarily involved in the underlying etiology. Due to their almost ubiquitous presence and ease of activation, clinical findings and epidemiology may suggest a causative role, even if the viruses are mere opportunists.

The delicate balance between latency and reactivation is designed by evolution. In a normal host, experiencing the normal interaction with the virus, the process is tuned to a long-term relationship that does not cause undue harm. However, if environmental factors upset this balance, or if the host for whatever reason is immunocompromised, the virus may inadvertently cause disease.

One might speculate that the optimal strategy for counteracting disease is to encourage early life exposure to herpesviruses. For the average person, infant inoculation with EBV and CMV may be beneficial, but the strategy does imply a risk for disease in rare individuals. Moreover, we live to an age where it is expected that the immune system has reduced potential, and occasionally we need to subdue the system in connection with various treatments. Thus, harmful consequences of these viruses are likely to occur at some point in life. One alternative is vaccines. We do have a vaccine for VZV, and vaccines are currently under development for HSV (49), EBV (50), and CMV (51). The more commonly used alternative at the present is medication. Fortunately, we have a considerable pharmaceutical arsenal to fight these viruses (9), unfortunately these medicines are unlikely to rid the body of virus.
